# An enChIP system for the analysis of genome functions in budding yeast

**DOI:** 10.1093/biomethods/bpac025

**Published:** 2022-10-17

**Authors:** Hodaka Fujii, Toshitsugu Fujita

**Affiliations:** Department of Biochemistry and Genome Biology, Hirosaki University Graduate School of Medicine, Hirosaki, Aomori 036-8562, Japan; Department of Biochemistry and Genome Biology, Hirosaki University Graduate School of Medicine, Hirosaki, Aomori 036-8562, Japan

**Keywords:** enChIP, dCas9, ChIP, chromatin immunoprecipitation, CRISPR, yeast, *Saccharomyces cerevisiae*

## Abstract

The identification of molecules associated with a specific genomic region is essential for elucidating the molecular mechanisms underlying genome functions such as transcription. Engineered DNA-binding molecule-mediated chromatin immunoprecipitation (enChIP) is a technology that enables the purification of specific genomic regions and the subsequent identification of their associated molecules. In enChIP, the target genomic region is tagged with engineered DNA-binding molecules, such as variants of the clustered regularly interspaced short palindromic repeats (CRISPR) system consisting of a catalytically inactive form of Cas9 (dCas9) and a guide RNA. This article describes the generation of a plasmid expressing *Streptococcus pyogenes* dCas9 fused to a 3xFLAG-tag (3xFLAG-Sp-dCas9) and its successful expression in the budding yeast, *Saccharomyces cerevisiae*. Furthermore, we showed that this plasmid can be used for enChIP analysis in budding yeast. In addition, the plasmid may also be a useful tool for researchers analyzing genome functions such as transcription and for CRISPR interference experiments in budding yeasts.

## Introduction

Identification of the regulatory molecules binding to a genomic region of interest is required to understand the molecular mechanisms underlying the regulation of genome functions, including transcription and epigenetic regulation. To achieve this goal, we developed engineered DNA-binding molecule-mediated chromatin immunoprecipitation (enChIP) technology, which enables isolation of a genomic region of interest and identification of its associated molecules [1]. Engineered DNA-binding molecules that can be used to tag a target locus include transcription activator-like proteins [2] and the clustered regularly interspaced short palindromic repeats (CRISPR) system [3–5], which consist of a catalytically inactive form of Cas9 (dCas9) and a guide RNA (gRNA) (see our recent reviews [6, 7] for comprehensive lists of publications using CRISPR-based systems). These engineered molecules can be expressed in a cell of interest to tag a locus (in-cell enChIP) [1] or incubated with fragmented chromatin *in vitro* (*in vitro* enChIP) [8]. *In vitro* enChIP utilizes a functional CRISPR complex consisting of recombinant dCas9 proteins and synthetic gRNA. The locus tagged with the engineered DNA-binding molecules is purified by affinity purification, and the associated molecules are identified by mass spectrometry (for proteins) [1, 9] or next-generation sequencing (for RNA and other genomic regions) [10–13].

This article describes the generation of a plasmid expressing *Streptococcus pyogenes* dCas9 fused to a 3xFLAG-tag (3xFLAG-Sp-dCas9) for use in enChIP analyses of the budding yeast, *Saccharomyces cerevisiae*. The plasmid will be useful for researchers in analyzing genome functions such as transcription by enChIP and may also be useful for analyses of gene functions through loss-of-function experiments using CRISPR interference (CRISPRi).

## Materials and methods

### Strains and media

The parental *S. cerevisiae* strain used in this study was YPH499 (*MATa*, *ura3-52*, *lys2-801*, *ade2-101*, *trp1-Δ63*, *his3-Δ200*, *leu2-Δ1*) (National Bio-Resource Project, BY18611) [14]. Yeast cells were grown in yeast extract–peptone–dextrose medium before transformation and were propagated in a synthetic-defined medium lacking tryptophan (SD/-TRP) or lakcing tryptophan and uracil (SD/-TRP/-URA) after transformation.

### Plasmids

To construct a plasmid expressing 3xFLAG-Sp-dCas9 in budding yeast (3xFLAG-dCas9/pTEF1p-CYC1t: Addgene, Watertown, MA, USA, Cat# 62190; RRID: Addgene_62190), the p414-TEF1p-Cas9-CYC1t plasmid (a gift from the George Church laboratory; Addgene, Cat# 43802; RRID: Addgene_43802) [15] was digested with SpeI (Takara Bio, Kusatsu, Japan, Cat# 1086A) and MluI (Takara Bio, Cat# 1071A), and treated with bacterial alkaline phosphatase (*Escherichia coli* C75) (Takara Bio, Cat# 2120A). A synthetic plasmid (14ACEQFP_1582159 QAD_3xFLAG-dCas9_ins_1-3) containing a synthesized double-stranded DNA (5′- actagtggatcccccgggaaaaATGGACTACAAAGACCATGACGGTGATTATAAAGATCATGACATCGATTACAAGGATGACGATGACAAGCTCATGGACAAGAAGTACTCCATTGGGCTCGCTATCGGCACAAACAGCGTCGGCTGGGCCGTCATTACGGACGAGTACAAGGTGCCGAGCAAAAAATTCAAAGTTCTGGGCAATACCGATCGCCACAGCATAAAGAAGAACCTCATTGGCGCCCTCCTGTTCGACTCCGGGGAGACGGCCGAAGCCACGCGGCTCAAAAGAACAGCACGGCGCAGATATACCCGCAGAAAGAATCGGATCTGCTACCTGCAGGAACTTGGGCGCGCCTGCAGCCTTCAAGTACTTCGACACCACCATAGACAGAAAGCGGTACACCTCTACAAAGGAGGTCCTGGACGCCACACTGATTCATCAGTCAATTACGGGGCTCTATGAAACAAGAATCGACCTCTCTCAGCTCGGTGGAGACAGCAGGGCTGACCCCAAGAAGAAGAGGAAGGTGTGAtctcttctcgagtcatgtaattagttatgtcacgcttacattcacgccctccccccacatccgctaaccgaaaaggaaggagttagacaacctgaagtctaggtccctatttatttttttatagttctatgttagtattaagaacgttatttatatttcaaatttttcttttttttctgtacagacgcgt-3′) (Thermo Fisher Scientific, Waltham, MA, USA) was also digested with SpeI and MluI. The cleaved vector and insert were purified after agarose gel electrophoresis and ligated. The resultant plasmid (3xFLAG-dCas9_E1 + 3/pTEF1p-CYC1t) was digested with SbfI (New England Biolabs, Ipswich, MA, USA, Cat# R642S) and AscI (Takara Bio, Cat# R0558S), and treated with bacterial alkaline phosphatase (*E. coli* C75). The dCas9 (SP)/pcDNA3.3 plasmid [1] was also digested with SbfI and AscI. The cleaved vector and insert were purified after agarose gel electrophoresis and ligated to generate the 3xFLAG-dCas9/pTEF1p-CYC1t plasmid. This plasmid contained the *TRP1* gene as a selectable marker, enabling the selection of transformed yeast cells in SD/-TRP. The p426-SNR52p-gRNA.CAN1.Y-SUP4t plasmid is a gift from the George Church laboratory; Addgene #43803, RRID: Addgene_43803) [15].

### Transformation of plasmids into YPH499

Transformation of 3xFLAG-dCas9/pTEF1p-CYC1t into yeast cells (65 ng/transformation) was carried out using a MicroPulser™ electroporator (Bio-Rad, Hercules, CA, USA, Cat# 1652100) according to a protocol for budding yeast provided by the manufacturer. After transformation, cells were plated on SD/-TRP medium and allowed to grow for 3 days at 30°C. Transformation of p426-SNR52p-gRNA.CAN1.Y-SUP4t into the yeast cells possessing 3xFLAG-dCas9/pTEF1p-CYC1t was performed by the same method, and cells were plated on SD/-TRP/-URA medium.

### Simple Western™ assay and general immunoblot analysis

Yeast cells were suspended in 100 µl (ca. 2.5 volumes of yeast pellet) of a crushing buffer (50 mM sodium phosphate, pH 7.4, 5% glycerol, 1 mM phenylmethylsulfonyl fluoride) and 40 µl of 0.5 mm glass beads (BioSpec Products, Bartlesville, OK, USA, Cat# 11079105), and were crushed with a Bead-Beater (BioSpec Products). Supernatants were recovered after centrifugation at 12,000 rpm for 15 min. Subsequently, 1.2 µg or 6.25 µg of the extract was subjected to Simple Western™ Assay using the “Wes” fully automated system (ProteinSimple, Bio-Techne, MN, USA) with an anti-FLAG M2 antibody (Ab) (Sigma-Aldrich, St Louis, MO, USA, Cat# F1804, RRID: AB_262044) or sodium dodecyl sulfate–polyacrylamide gel electrophoresis (SDS–PAGE) followed by Coomassie Brilliant Blue (CBB) staining, respectively. Alternatively, the extract (20 µg) was subjected to a general immunoblot analysis with an anti-FLAG M2 Ab as described previously [1] or SDS–PAGE followed by CBB staining.

### enChIP

Yeast cells (ca. 2 × 10^8^ cells) were cultured and fixed with 1% formaldehyde. After quenching with 125 mM glycine, yeast cells were collected and lysed in 800 µl of Lysis buffer [0.1% sodium deoxycholate, 1 mM ethylenediaminetetraacetic acid (EDTA), 50 mM 4-(2-hydroxyethyl)-1-piperazineethanesulfonic acid (HEPES)-KOH, pH7.5, 140 mM NaCl, 1% Triton X-100, and cOmplete protease inhibitor cocktail without EDTA (Roche, Basel, Switzerland)] containing 0.5 mm glass beads, and crushed with a Bead-Beater. Chromatin DNA in supernatants was fragmented by sonication with a Smurt NR-50M (Microtec, Chiba, Japan) using the following parameters: 25% power output, 10 cycles of 30 s ON, and 60 s OFF, on ice. After centrifugation at 12,000 rpm for 15 min, a part of the supernatant (a volume equivalent to 4 × 10^6^ cells) was subjected to enChIP followed by quantitative PCR (qPCR) analysis (enChIP-qPCR). enChIP-qPCR was performed as described previously [1]. Primers used in this study were as follows: gRNA_CAN1Y_SC_Y12-F_28924 (5′-tcagcgttctgtacttctccttc-3′) and gRNA_CAN1Y_SC_Y12-R_28925 (5′-aattgtatccattgcgctcttt-3′) for the *CAN1* locus, gRNA_ADE2Z_SC_Y12-F_28928 (5′-aggaacatcaacatgctcaatct-3′) and gRNA_ADE2Z_SC_Y12-R_28929 (5′-aaataagcaactccaatgaccac-3′) for the *ADE2* locus.

## Results and discussion

**Figure 1:**
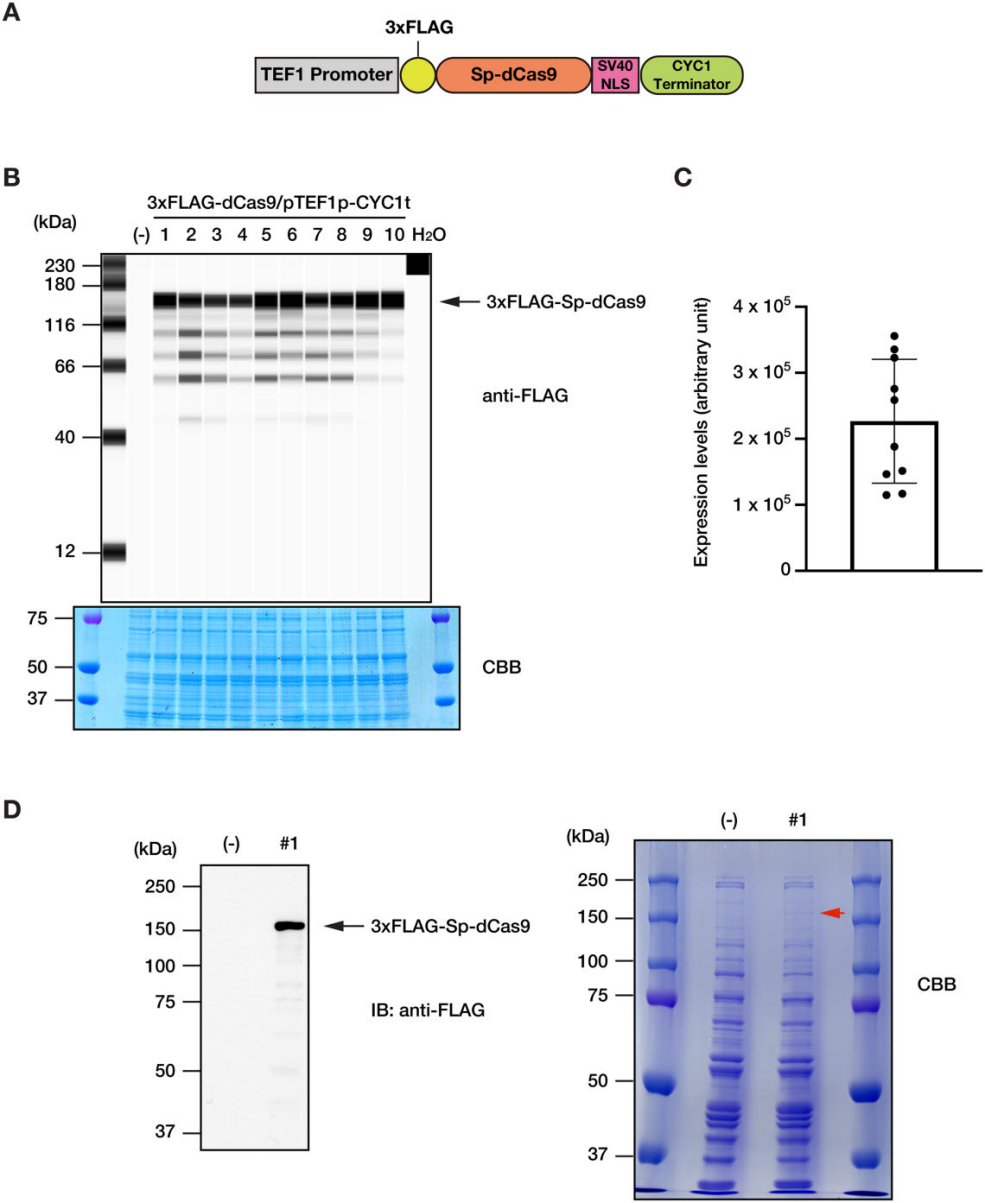
Expression of 3xFLAG-Sp-dCas9 from the 3xFLAG-dCas9/pTEF1p-CYC1t plasmid in budding yeast. (**A**) Schematic illustration of the 3xFLAG-dCas9/pTEF1p-CYC1t plasmid expressing 3xFLAG-Sp-dCas9. The *TEF1* constitutive promoter drives transcription of 3xFLAG-dCas9 fused with the SV40 nuclear localization signal (NLS) and the CYC1 terminator. (**B**) Expression of 3xFLAG-Sp-dCas9, detected via Simple Western™ assay with an anti-FLAG Ab, in 10 randomly selected YPH499 clones transformed with the 3xFLAG-dCas9/pTEF1p-CYC1t plasmid. CBB staining is shown as a protein loading control. (-): the parental YPH499 strain. (**C**) Quantification of expression levels of 3xFLAG-Sp-dCas9 in the selected clones. Background noise was subtracted from each signal. Standard deviation is shown. (**D**) Expression of 3xFLAG-Sp-dCas9 in a representative clone (#1). Expression of 3xFLAG-Sp-dCas9 was detected via immunoblotting with an anti-FLAG Ab (left panel). CBB staining is shown as a protein loading control (right panel). An extra band corresponding to 3xFLAG-Sp-dCas9 is visible in the 3xFLAG-dCas9/pTEF1p-CYC1t-transformed clone (#1) (arrowhead) (-): the parental YPH499 strain.

To perform analyses using the dCas9 protein in budding yeast, we constructed the 3xFLAG-dCas9/pTEF1p-CYC1t plasmid, in which the expression of 3xFLAG-Sp-dCas9 is driven by the *TEF1* promoter (Fig. 1A). The plasmid contains an autonomous replication origin (CEN/ARS). Transformed yeast cells can be selected in a tryptophan-deficient medium.

The 3xFLAG-dCas9/pTEF1p-CYC1t plasmid was transformed into the *S. cerevisiae* YPH499 strain, and transformants were selected on SD/-TRP plates. The parental YPH499 strain did not grow on this medium, whereas many colonies were observed for transformants.

Expression of 3xFLAG-Sp-dCas9 was analyzed in 10 randomly selected clones. As shown in Fig. 1B and C, all 10 clones showed comparable levels of 3xFLAG-Sp-dCas9, suggesting that the 3xFLAG-dCas9/pTEF1p-CYC1t plasmid is stably retained in this budding yeast strain. These results showed that the transformation efficiency in budding yeast for successful expression of 3xFLAG-Sp-dCas9 is very high (100%). Figure 1D shows an immunoblot analysis and CBB staining of a representative clone (#1). A closer look at the CBB staining image revealed that an extra band of ca. 160 kDa was visible (Fig. 1D, arrowhead in the right panel), suggesting that the expression level of 3xFLAG-Sp-dCas9 in this strain was relatively high.

**Figure 2:**
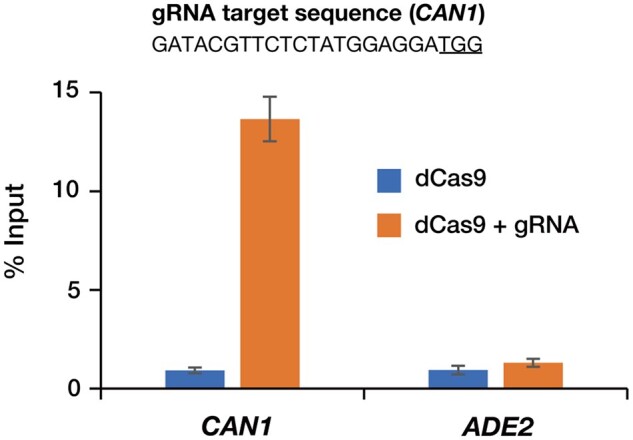
Isolation of the *CAN1* locus by enChIP. One of the YPH499 clones expressing 3xFLAG-Sp-dCas9 was further transformed with the p426-SNR52p-gRNA.CAN1.Y-SUP4t plasmid expressing gRNA targeting the *CAN1* locus. After enChIP, qPCR was performed to analyze the yield of the target locus (*CAN1*). An irrelevant locus (*ADE2*) was analyzed as a negative control. Error bars represent the standard deviation [*n* = 3 (dCas9) or 4 (dCas9 + gRNA)]. The underline in the gRNA target sequence represents the protospacer adjacent motif (PAM) site.

After deposition of 3xFLAG-dCas9/pTEF1p-CYC1t at Addgene a few years ago, the coding sequence of 3xFLAG-Sp-dCas9 in this plasmid was successfully used for enChIP and other analyses in SK1-derived budding yeast strains [16]. We also succeeded in the locus-specific isolation of a target region when an enChIP analysis was performed with a gRNA targeting the *CAN1* gene expressed by transformation with the p426-SNR52p-gRNA.CAN1.Y-SUP4t plasmid [15] (Fig. 2). It is expected that 3xFLAG-dCas9/pTEF1p-CYC1t could also be used to express 3xFLAG-Sp-dCas9 for other applications such as those employing CRISPRi.

## Conclusions

This report describes the generation of 3xFLAG-dCas9/pTEF1p-CYC1t, an expression plasmid harboring 3xFLAG-Sp-dCas9, and its stable retention in a budding yeast strain. Expression of the 3xFLAG-Sp-dCas9 protein was confirmed by Simple Western™ assay, immunoblot analysis, and CBB staining. Furthermore, we showed that this plasmid can be used for enChIP analysis in budding yeast. This plasmid will be useful for enChIP analyses and other applications in budding yeast.

## Limitations

This report used only a single budding yeast strain, YPH499. It would also be useful to determine whether the 3xFLAG-Sp-dCas9 protein can be expressed from 3xFLAG-dCas9/pTEF1p-CYC1t in other yeast strains. However, since YPH499 is not exceptional in terms of its regulation of transcriptional and translational machineries and other physiological properties [14], it is reasonable to assume that 3xFLAG-dCas9/pTEF1p-CYC1t could indeed be used successfully in other strains. In addition, we did not examine whether the 3xFLAG-Sp-dCas9 protein is expressed when the plasmid is integrated into the chromosome although we do not see why an integrated form of the plasmid would not support expression of 3xFLAG-Sp-dCas9. In this regard, such use has several merits; for example, it would not be necessary to continuously select plasmid-positive cells with selection media. On the other hand, it would be necessary to select multiple clones to obtain one with adequate levels of expression because plasmids randomly integrate into chromosomes, and gene expression is influenced by the genome environment near the integration sites. Although continuous selection is necessary to maintain yeast clones bearing plasmids, the establishment of plasmid-positive clones is easy, as shown in this manuscript, and the different clones showed comparable expression levels of 3xFLAG-Sp-dCas9. Therefore, such a strategy would be convenient for most, but not all, research applications.

## Data Availability

All data generated or analyzed from the current study are included in this published article.
